# Factor structure and sex invariance of the temporal experience of pleasure scale (TEPS) in Chinese university students and clinical population

**DOI:** 10.1186/s12888-021-03379-9

**Published:** 2021-07-28

**Authors:** Shulin Fang, Xiaodan Huang, Panwen Zhang, Jiayue He, Xingwei Luo, Jianghua Zhang, Yan Xiong, Fusheng Luo, Xiaosheng Wang, Shuqiao Yao, Xiang Wang

**Affiliations:** 1 Medical Psychological Center, the Second Xiangya Hospital,Central South University, Changsha, 410011 China; 2 Student Affairs Department, Central South University, Changsha, 410083 China; 3 Student Affairs Department, Central South University of Forestry and Technology, Changsha, 410004 China; 4 Department of Human Anatomy and Neurobiology, Xiangya School of Medicine, Central South University, Changsha, 410013 China; 5 Medical Psychological Institute of Central South University, Changsha, 410011 China; 6 National Clinical Research Center for Mental Disorders, Changsha, 410011 China

**Keywords:** Psychometric properties, Anhedonia, Motivation, Exploratory factor analysis, Confirmatory factor structure

## Abstract

**Background:**

A motivation dimension of the core psychiatric symptom anhedonia additional has been suggested. The Temporal Experience of Pleasure Scale (TEPS) has been reported to assess anticipatory and consummatory pleasure separately in multiple factor-structure models. This study explored the factor structure of a Chinese version of the 18-item TEPS and further explored the measurement invariance of the TEPS across sex and clinical status (non-clinical, psychiatric).

**Methods:**

Best-fit factor structure of the TEPS was examined in a non-clinical cohort of 7410 undergraduates, randomized into sample 1 (*N* = 3755) for exploratory factor analysis (EFA) and sample 2 (*N* = 3663) for confirmatory factor analysis (CFA). Additionally, serial CFA was conducted to evaluate measurement invariance across sex and between clinical (*N* = 313) and non-clinical (*N* = 341) samples.

**Results:**

EFA supported a new four-factor structure with a motivation component, based on the original two-factor model (consummatory pleasure with/without motivation drive, anticipatory pleasure with/without motivation drive). CFA confirmed the four-factor model as the best-fit structure and revealed a second-order hierarchy in non-clinical and clinical samples. Full scalar invariance was observed across clinical and non-clinical samples and across sex in the clinical sample; only partial scalar invariance was observed across sex in the non-clinical sample.

**Conclusions:**

A four-factor structured TEPS can assess motivation-driving dimensions of anticipatory and consummatory pleasure, consistent with the recently advanced multidimensional structure of anhedonia. CFA and measurement invariance results support application of the TEPS for assessing motivation aspects of anhedonia.

**Supplementary Information:**

The online version contains supplementary material available at 10.1186/s12888-021-03379-9.

## Background

Anhedonia, defined as a diminished or absent ability to experience pleasure [1], is a core symptom of several psychiatric disorders, most prominently including major depressive disorder (MDD) and schizophrenia [2–4]. In recent years, anhedonia has been linked to the increased risk of many other kinds of neuropsychiatric diseases, for example, Vaquero-Puyuelo D et al. found that anhedonia is a risk factor for Alzheimer’s disease in a longitudinal study [5]. Several scales aimed at evaluating anhedonia have been developed. Some initial measurements, such as the Fawcett-Clark Pleasure Capacity Scale [6] and the Snaith-Hamilton Pleasure Scale [7], developed in the early 1980s and 1990s, respectively, treat anhedonia as a unitary construct. Meanwhile, research providing deeper insights into anhedonia has indicated that anhedonia should be considered as a complex, multidimensional concept with relations to multiple psychopathological processes, including processes in physical/social, consummatory/anticipatory, and motivation/experiential dimensions [8, 9]. Scales with a focus on the physical/social dimension, including the Revised Physical Anhedonia Scale and the Revised Social Anhedonia Scale, have been used to assess anhedonia in patients with schizophrenic spectrum disorders and in other clinical samples since the 1970s [10, 11].

In the last two decades, there has been a growing acceptance of the concepts of anticipatory and consummatory anhedonia based on a convergence of evidence from animal and human studies [12–14]. In response to this evolution in the field, Gard and colleagues developed the Temporal Experience of Pleasure Scale (TEPS), which was designed to reflect anticipatory and consummatory anhedonia [15]. In response to a recent growing emphasis on motivational aspects of anhedonia existing beyond the consummatory/anticipatory dimension [16, 17], the Motivation and Pleasure Scale (self-report) was developed to assess motivational anhedonia [18]. Although several scales assess anhedonia from a particular dimension perspective, a scale that encompasses the multi-dimensionality of anhedonia comprehensively has been lacking.

The TEPS has distinct advantages with respect to assessing anticipatory and consummatory anhedonia, and has been supported by substantial preclinical and nonclinical studies [19, 20]. It has also been translated into multiple language versions and demonstrated to be a reliable and valid scale for the measurement of anhedonia [21–24]. However, there is inconsistency among studies with respect to the factor structure of the TEPS. The initial TEPS validation study reported by Gard and colleagues, which was conducted with a sample of American university students, yielded a two-factor structure with anticipatory and consummatory factors [15]. Subsequent studies of the French and German versions, conducted in both healthy controls and schizophrenic patients, replicated the two-factor structure proposed in the initial study [21–23]. However, the Italian and Persian versions were found to have three-factor structures wherein it appeared to be more suitable to subdivide anticipatory anhedonia into contextual and abstract anticipatory anhedonia [25, 26]. In 2012, Chan et al. developed a Chinese version of the TEPS that included two additional items, altering the scale from an 18-item to a 20-item version, and then reported the psychometric properties of the new 20-item version in a non-clinical sample of undergraduate students [24]. Based on their findings, they proposed a four-factor structure for their 20- item TEPS, with the factors being consummatory contextual, consummatory abstract, anticipatory contextual, and anticipatory abstract [27]. The addition of two items, however, made it difficult to compare the properties found for the 20-item version with other international studies of 18-item versions of the TEPS. Moreover, it seems that the abstract-contextual dimension divisions were made primarily to suit to the anticipatory pleasure construct, with less relevance to the consummatory pleasure construct. For example, the “abstract” consummatory factor items may also refer to specific aspects of contextual pleasure (e.g. item 2: The sound of crackling wood in the fireplace is very relaxing). Therefore, the factor structure and interpretation of the Chinese TEPS, particularly of the original 18-item version, needs further exploration in non-clinical and clinical samples.

Despite debates regarding its factor structure, the TEPS has continued to be used worldwide in patients with psychiatric disorders, and has been reported to have good reliability and validity for assessing degrees of consummatory and anticipatory anhedonia [21, 23, 28]. For instance, in a schizophrenia sample, an 18-item German version of the TEPS was found to have a Cronbach’s α of 0.85 for the full scale, and scores correlated with scores from self-rated anhedonia and clinician-rated apathy measures [23]. Interestingly, impairments in consummatory pleasure and anticipatory pleasure appear to be distinguishable and distinct across different psychiatric disorders. Schizophrenics have been found to have reduced anticipatory pleasure, compared to healthy controls, but an intact capacity for consummatory pleasure [20, 29, 30], whereas patients with obsessive-compulsive disorder have reduced consummatory pleasure with intact anticipatory pleasure [31], and patients with MDD have reduced anticipatory and consummatory pleasure [32].

The aforementioned studies indicate that anhedonia is a multidimensional construction and that understanding the pathology of anhedonia involves distinguishing among its components. However, the question of how these differences may contribute to authentic differences between clinical and non-clinical samples remains to be clarified. Furthermore, there remains a need for analyses of the measurement invariance of the TEPS across different populations. Measurement invariance, which is an index of the stability of the meanings of scale items and factor structure across different populations, can be determined through a series of confirmatory factor analyses (CFAs) with increasingly constrained conditions enabling researchers to confirm that differences observed between groups reflect true differences between groups rather than an artifact of the scale being used [33].

Sex differences in anhedonia have also been reported between different psychiatric disorder populations. For example, schizophrenic male score higher than schizophrenic female on physical anhedonia and social anhedonia measures [34]. Additionally, trait anhedonia in female, but not male, alcoholics can predict depressiveness at the end of detoxification [35]. Although measurement invariance across sex has been reported for the TEPS in a healthy population sample [27], measurement invariance of the TEPS across sex in populations with psychiatric disorders has not been established. Such measurement invariance is important for the development of individualized clinical treatment plans in light of differing anhedonia scale findings between males and females.

The present study pursued four aims. Firstly, the reliability including internal consistency reliability and retest reliability of 18-item version of TEPS were explored in this study. Secondly, we used combined exploratory factor analysis (EFA) and CFA to determine the best-fit factor structure of a Chinese version of the 18-item TEPS in non-clinical and clinical samples. Thirdly, we explored the psychological mechanism of anhedonia revealed by that factor structure. Additionally, we used the best-fit factor structure to probe measurement invariance across clinical and non-clinical samples and across sex groups.

## Methods

### Sample and procedure

We recruited 7418 college students from two universities in Hunan Province to take part in this study and randomized them into two samples: one used for EFA to probe the factor structure of the TEPS (N_sample1_ = 3755); and the other used for CFA (N_sample2_ = 3663). Sample 1 consisted of 1987 (52.9%) males and 1768 (47.1%) females. Sample 2 consisted of 1939 (52.9%) males and 1724 (47.1%) females. The two samples had similar mean TEPS total scores [t = 0.660, degrees of freedom (df) = 7415, *p* = 0.511]. A subset of 312 college students (72 males, 23.1%; and 240 females, 76.9%) were selected randomly for retesting 4 weeks later.

Sample 3 was constituted by 313 psychiatric outpatients (158, 50.5% males; and 155, 49.5% females) from the second Xiangya Hospital who had been diagnosed by two psychiatrists according to the fourth Diagnostic and Statistical Manual of Mental Disorder (DSM-IV). This sample included 94 outpatients diagnosed with major depressive disorder (MDD), 29 outpatients diagnosed with schizophrenia, and 190 outpatients diagnosed with a personality disorders (obsessive-compulsive disorder, schizotypal personality disorder, among others). Sample 4 consisted of 341 healthy control individuals (171, 50.1% males; and 170, 49.9% females) recruited from the university and surrounding community to be demographically similar to Sample 3.

We used posters and advertisements to recruit participants from universities, communities, and hospitals. The group-administeredpaper-pencil measure was taken to collect data from volunteered participants with two well-trained psychological postgraduate researchers’ guidance in a quiet room. All questionnaires were returned immediately after participants accomplish and were checked whether existing missing items. We choose group-administered measure in sample1, sample2, and sample 4, while single-administered measure in sample 3. All participants provided written informed consent.

### Instrument

The standard TEPS is a self-report questionnaire designed to assess anhedonia severity in adolescents and adults. It consists of 18 items, each of which is rated from 1 (very false for me) to 6 (very true for me); item 7 is reverse coded. Total TEPS scores range from 18 to 108, with a lower score reflecting a greater severity of anhedonia. The TEPS has been demonstrated to have excellent reliability and discrimination validity in various samples, and our initial research showed an internal consistency reliability is 0.79 and retest reliability is 0.81 [15]. In this study, we report mean (*M*) TEPS scores with standard deviations (SDs).

A Chinese version of the 18-item TEPS was developed according to the second edition of International Test Commission (ITC) Guidelines for Translating and Adapting [36], which includes four steps to obtain a credible translated and culturally adapted instrument. Firstly, permission for the translation and adaption of the TEPS in China was obtained from the initial original creators of the TEPS. Secondly, two psychologists translated the original English-language TEPS to Chinese with full consideration of Chinese culture in the process of translation. Thirdly, another bilingual expert who was unaware of the original version translated the Chinese version to English and then comparing and adjusting for contradictions between the original English version and the back-translated English version. Finally, we evaluated the scale in ten undergraduate students and according to their feedback to conduct the final adjustment, and determine the final Chinese version of TEPS.

### Data analysis

#### Reliability

Three reliability coefficients including Cronbach’s α, mean inter-item correlation (MIC) values, and McDonald’s omega were used to assess the internal reliability of the TEPS in all four samples. For detail, Cronbach’s α above 0.70 can acceptable [37], the optimal MIC ranged 0.10–0.40 [38], and the McDonald’s omega above 0.70 can acceptable [39]. The retest reliability was assessed with Spearman correlation analysis [40].

#### EFA

EFA to probe the adaptive factor structure of the TEPS was conducted with Sample 1. First, we applied the traditional criterion to determine the number of factors to retain, wherein the eigenvalues-greater-than-one rule was applied [41, 42]. Second, we conducted parallel analysis in *M-plus* (version 7.0) [43] based on a comparison between eigenvalues from a factor analysis of the actual data and eigenvalues from a factor analysis of a random dataset (1000 random permutations of the original data); the number of factors retained was based on the number of actual data eigenvalues in the upper 95% confidence limit of the random data eigenvalues [44, 45]. Third, Velicer’s minimum average partial (MAP) test was conducted in SPSS (version 25.0, IBM, 2017) to determine the number of factors to retain. The MAP test is focused on the relative amounts of systematic and non-systematic variance remaining in a correlation matrix after extractions of increasing numbers of components [46, 47]. Finally, we considered the variance of the outputs of these methods (eigenvalues-greater-than-one rule, parallel analysis, and MAP test), and compared the model fits of solutions with different numbers of factors obtained from the above methods in *M-plus* (version 7.0)[43].

This study used a maximum likelihood with robust standards errors (MLR) method rather than the maximum likelihood method to extract factors because the latter requires data to be normally distributed, which was too restrictive for the present study, and because MLR is an optimal choice even for normally distributed data owing to its yielding the best combination of accurate standard errors and Type 1 errors [48]. Oblique rotation was used because of the interrelatedness of the factors.

#### CFA

A series of CFAs were conducted to compare two previously proposed structure models in the literature (the Gard model and the Chan model) with the factor model developed in this study with Samples 2. The Gard model has a two-factor structure, with a consummatory pleasure factor (items 2, 4, 5, 6, 8, 9, 10, 13) and a anticipatory pleasure factor (items 1, 3, 7, 11, 12, 14, 15, 16, 17, 18) [15]. The Chan model has a four-factor structure, including contextual consummatory pleasure (items 2, 3, 10, 17), abstract consummatory pleasure (items 4, 5, 6, 8, 13), contextual anticipatory pleasure (items 9, 11, 12, 14, 18), and abstract anticipatory pleasure (items 1,15,16) factors. The two additional items added by Chan and colleagues were attributed to the contextual anticipatory (item 19) and abstract consummatory (item 20) factors [24], and item 7 was omitted. The best fit factor model according to our EFA results was accepted as a final model. Overall, three confirmatory factor analyses were conducted in this study: Gard model (two-factor structure,18 items), Chan model (four-factor structure, 20 items), and new factor structure found in this study according to results of EFA analysis (18 items). The following model fit indices were applied: Tucker-Lewis index (TLI) ≥ 0.90, comparative fit index (CFI) ≥ 0.90, root mean square error of approximation (RMSEA) ≤ 0.08, and the standardized root mean square residual (SRMR) ≤ 0.08 [49–51].

#### Convergent validity and discriminate validity

The convergent validity and discriminate validity of the best fit factor structure obtained from the results of EFA and CFA were assessed in sample 2. The composite reliability (CR) was used to represent the convergent validity in this study and the cut-off criteria is set above 0.60 [52]. The hetereotrait-monotrait ratio (HTMT) of correlations was used to represent the discriminate validity and the cut-off criteria is set below 0.85 [53].

#### Measurement invariance

Sample 3 (clinical) and Sample 4 (non-clinical) were used to probe measurement invariance across clinical and non-clinical samples. Samples 1 and 2 were used together to assess measurement invariance across sex in a non-clinical sample and Sample 3 was used to assess measurement invariance across sex in a clinical sample. The final model obtained by CFA was applied to these measurement invariance analyses. Four measurement invariance models with increasing cross-group restrictions on parameters were applied: (1) configural invariance, which tests factor structure invariance of factor latent variables across groups; (2) metric invariance, which tests factor loading invariance across groups; (3) scalar invariance, which tests intercept invariance across groups; and (4) strict invariance, which tests error variance invariance across groups [54]. In the event of failed metric or scalar invariance, indices need to be modified to determine items in which factor loadings and intercepts failed to reach invariance, and constrictions for these items need to be relaxed.

CFI and RMSEA differences between increasingly constrained models, termed ΔCFI and ΔRMSEA, were used to evaluate model suitability for model confirmation. The criteria for acceptable invariance were ΔCFI ≤0.01, ΔRMSEA ≤0.01 and smaller Bayesian information criterion (BIC) [54, 55].

## Results

### Descriptive statistics and reliability

In Sample 1, TEPS total scores ranged from 26.00 to 108.00 (77.57 ± 12.30), with a Cronbach’s α, McDonald’s omega, and MIC values of 0.83, 0.84, and 0.22, respectively. In Sample 2, TEPS total scores ranged from 23.00 to 108.00 (77.38 ± 12.60), with a Cronbach’s α, McDonald’s omega, and MIC value of 0.84, 0.85, and 0.23, respectively. In Sample 3, TEPS total scores ranged from 22.00 to 108.00 (71.35 ± 15.86), with a Cronbach’s α, McDonald’s omega, and MIC value of 0.87,0.88, and 0.28, respectively. In Sample 4, TEPS total scores ranged from 29.00 to 108.00 (74.62 ± 12.96), with a Cronbach’s α, McDonald’s omega, and MIC value of 0.84,0.85, and 0.23, respectively. The retest reliability of the total scale calculated by Spearman correlation analysis was 0.70 (*p* < 0.05). Compared to the non-clinical Sample 4, the clinical Sample 3 had lower TEPS scores (t = 2.869, df = 603.60, *p* = 0.004, Cohen’s d = 0.23), which indicated that clinical sample 3 showed more serious degree of anhedonia.

### EFA of the structure of the TEPS

The eigenvalues-greater-than-one rule suggested we retain four factors, MAP testing suggested we retain two factors, and parallel analysis suggested we retain three factors. Comparing the model fit indices of these three different solutions which showed in Table 1, we found that the four-factor structure was the best solution (factor loadings in Table 2) and thus retained the four-factor structure.

**Table 1:** Goodness-of-fit indices of two-, three-, and four-factor solutions for the structure of the Chinese 18-item TEPS based on EFA

Model	X^2^	*df*	SRMR	CFI	RMSEA (90%CI)
Two factors	1669.629	118	0.039	0.864	0.059 (0.057 0.062)
Three factors	924.218	102	0.028	0.928	0.046 (0.044 0.049)
Four factors	609.930	87	0.022	0.954	0.040 (0.037 0.043)

*Note.* X^2^, Chi-square; df, degrees of freedom; CFI, comparative fit index; RMSEA, root mean square error of approximation

**Table 2:** Factor loading of retained four-factor structure of the TEPS based on EFA

			Factor loading			
			1	2	3	4
**Factor 1: consummatory pleasure without motivation-driving**						
2.The sound of crackling wood in the fireplace is very relaxing			**0.42**	−0.04	−0.07	0.15
4.I love the sound of rain on the windows when I’m lying in my warm bed			**0.55**	−0.05	−0.04	0.19
5.The smell of freshly cut grass is enjoyable to me			**0.65**	−0.01	0.03	0.04
6. I enjoy taking a deep breath of fresh air when I walk outside			**0.56**	0.04	0.24	−0.10
8.A hot cup of coffee or tea on a cold morning is very satisfying to me			**0.35**	0.13	0.16	0.04
13. I appreciate the beauty of a fresh snowfall			**0.39**	0.15	0.21	−0.05
**Factor 2: consummatory pleasure with motivation-driving**						
9. I love it when people play with my hair			0.19	**0.32**	−0.06	0.05
10. I really enjoy the feeling of a good yawn			0.23	**0.40**	−0.01	0.00
11. When I’m on my way to an amusement park, I can hardly wait to ride the roller coasters			0.00	**0.75**	0.05	0.01
12. I get so excited the night before a major holiday I can hardly sleep			−0.05	**0.44**	0.00	0.19
**Factor 3: anticipatory pleasure without motivation-driving**						
1.When something exciting is coming up in my life, I really look forward to it			0.12	0.17	**0.31**	0.05
15.Looking forward to a pleasurable experience is in itself pleasurable			0.12	0.02	**0.55**	0.14
16. I look forward to a lot of things in my life			−0.03	−0.05	**0.89**	0.02
**Factor 4: anticipatory pleasure with motivation-driving**						
3.When I think about eating my favorite food, I can almost taste how good it is			0.23	−0.02	0.04	**0.49**
14.When I think of something tasty, like a chocolate chip cookie, I have to have one			0.05	0.16	0.00	**0.50**
17.When ordering something off the menu, I imagine how good it will taste			−0.03	0.06	0.13	**0.60**
18. When I hear about a new movie starring my favorite actor, I can’t wait to see it			0.00	0.17	0.02	**0.39**
7. I don’t look forward to things like eating out at restaurants (R)			−0.19	0.13	0.14	0.18
Factor correlations						
	Factor 1	Factor 2	Factor 3	Factor 4		
Factor 1	1.00					
Factor 2	0.28	1.00				
Factor 3	0.31	0.46	1.00			
Factor 4	0.25	0.55	0.46	1.00		

*Note:* R, reverse-coded; Factor loadings above 0.30 are in bold; Based on low factor-loading and reverse coding, item 7th was omitted when confirming the new four-factor structure and subsequent measurement invariance analyses

The item composition of the retained four-factor structure was as follows: factor 1, items 2, 4, 5, 6, 8, and 13; factor 2, items 9, 10, 11, and 12; factor 3, items 1,15, and 16; and factor 4, items 3, 7, 14, 17, and 18. Analyzing item contents, we found that it was possible to subdivide the consummatory pleasure and anticipatory pleasure factors into with and without motivation dimensions, generating consummatory pleasure with/without motivation-driving subscales as well as anticipatory pleasure with/without motivation-driving subscales. Items in the with motivation-driving subscales reflects motivation to take action to satisfy a desire, whereas those in the without motivation-driving subscale represent in-the-moment enjoyment (consummatory) or the expectation to enjoy a pleasure (anticipatory) without any motivation to take action.

### CFA of the structure of the TEPS

**Fig. 1:**
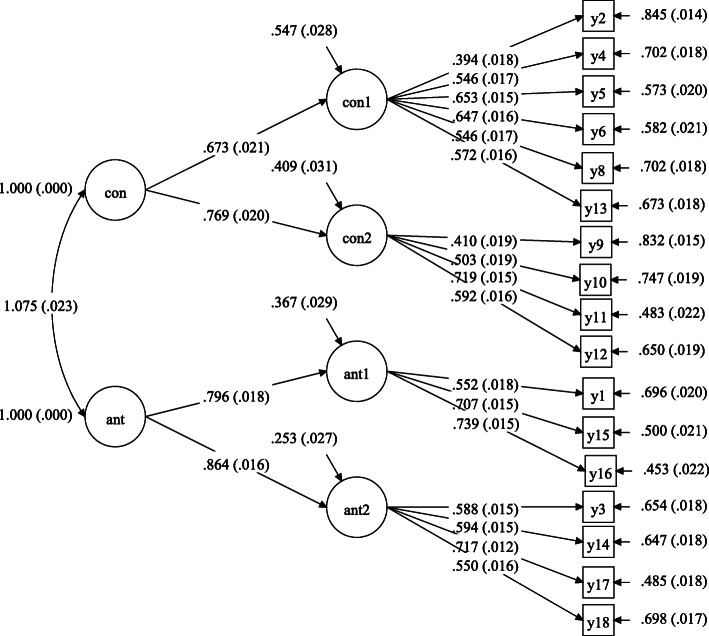
Second-order model of Chinese version of the 18-item TEPS in sample 2(undergraduate sample). *Note*: Con, consummatory pleasure; Ant, anticipatory pleasure; Con1, consummatory pleasure without motivation driving; Con 2, consummatory pleasure with motivation driving; Ant1, anticipatory pleasure without motivation driving; Ant 2, anticipatory pleasure with motivation driving; y1 to y18 means item1 to item 18

As shown in Table 3, CFA indicated that our newly explored structure had a better fit factor structure (i.e. better fit index values) than Gard’s and Chan’s models, both in our non-clinical undergraduate sample and our clinical sample. Follow-upsecond-level CFA from a consummatory-anticipatorysecond-order view indicated that the second-level model fits well in both samples (Table 3). In addition, the Second-order model of the Chinese version of the 18-item TEPS both in undergraduate students can reference Fig. 1. Meanwhile, this study also conducted CFAs in the clinical sample 3 to test whether factor structure found in university students is equally in clinical sample, the detailed results is shown in Table S1 in supplementary material.

**Table 3:** Goodness-of-fit indices obtained for compared structural models of the TEPS in undergraduate sample (sample 2)

	X^2^	*df*	CFI	TLI	SRMR	RMSEA (90%CI)
Model1	2110.259	134	0.830	0.806	0.053	0.063 (0.061 0.066)
Model2	1392.040	113	0.886	0.863	0.046	0.056 (0.053 0.058)
Model3	1121.298	113	0.910	0.892	0.043	0.049 (0.047 0.052)
Second-level	1189.219	114	0.904	0.886	0.044	0.051 (0.048 0.053)

*Note:* Model 1 is the two-factor structure proposed by Gard (18 items and two factor structure) [15]. Model 2 is the four-factor structure proposed by Chan [24] with two added items, and item 7 regarded as expendable (20 items and four factor structure). Model 3 is our newly developed four-factor structure without item 7(18 items and four factor structure). X^2^, Chi-square; df, degrees of freedom; CFI, comparative fit index; TLI, Tucker-Lewis index; SRMR, standardized root mean squared residual; RMSEA, root mean square error of approximation

### Convergent validity and discriminate validity

For the convergent validity, the CR for the consummatory pleasure with/without motivation driving and anticipatory pleasure with/without motivation driving is 0.65, 0.73, 0.71 and 0.71 respectively, all above 0.60 that showed the new four-factor structure found in this study with good convergent validity. For the discriminate validity, as shown in Table 4, the HTMT ranged from 0.59 to 0.78, all below 0.85 that showed the new four-factor structure found in this study with good discriminate validity.

**Table 4:** The Heterotrait-Monotrait ratio of correlations (HTMT) in undergraduate sample (sample2)

	Con1	Con2	Ant1	Ant2
Con1				
Con2	0.59			
Ant1	0.65	0.62		
Ant2	0.60	0.78	0.70	

*Note:* Con1, consummatory pleasure without motivation driving, Con2, consummatory pleasure with motivation driving; Ant1, anticipatory pleasure without motivation driving; Ant2, anticipatory pleasure with motivation driving

### Measurement invariance

Fit indices for configural invariance indicated that our newly developed four-factor structure of latent variables is invariant across clinical and non-clinical samples, thus establishing our baseline model. CFI and RMSEA differentials between the configural model and metric model were all < 0.01, affirming metric invariance across the clinical and non-clinical samples. Scalar invariance was also established based on meeting the mutative index criteria. Lastly, strict invariance was established based on our obtained ΔCFI and ΔRMSEA values being < 0.01. The data obtained in our measurement invariance analyses across clinical and non-clinical samples, from configural invariance to strict invariance, are reported in Table 5.

**Table 5:** Measurement invariance across clinical and non-clinical samples based on CFA

	X^2^	*df*	CFI	BIC	SRMR	RMSEA (90%CI)	ΔCFI	ΔRMSEA
configural	418.548	226	0.922	37,843.902	0.054	0.051 (0.043 0.059)		
metric	444.234	239	0.917	37,785.676	0.061	0.051 (0.044 0.059)	−0.005	0.000
scalar	470.730	252	0.911	37,728.430	0.062	0.052 (0.044 0.059)	−0.006	+ 0.001
strict	508.677	269	0.903	37,661.713	0.065	0.052 (0.045 0.059)	−0.007	0.000

*Note:* Measurement invariance across clinical and non-clinical samples analysis based on our newly explored structure (17 items and four factor structure). Model a, configural invariance; Model b, metric invariance; Model c, scalar invariance; Model d, error variance invariance. X^2^, Chi-square; *df*, degrees of freedom; CFI, comparative fit index; RMSEA, root-mean-square error of approximation; SRMR, standardized root mean squared residual; BIC, Bayesian information criterion

As shown in Table 6, partial scalar invariance across sex was obtained in a non-clinical sample (Samples 1 + 2) under the condition that permitting free intercept estimations of items 2, 9, 11, and 18, based on modification indices. CFA of the new four-factor structure indicated a slight insufficiency in Sample 2; thus, freely estimated error covariance between items 2 and 4 was permitted when conducting measurement invariance across sex in the non-clinical sample. In the clinical sample (Sample 3), full measurement invariance analysis yielded ΔCFIs < 0.01 and ΔRMSEAs < 0.001 between increasingly constrained models.

**Table 6:** Measurement invariance across sex based on CFA

Model	X^2^	*df*	CFI	BIC	SRMR	RMSEA (90%CI)	ΔCFI	ΔRMSEA
**Non-clinical**								
configural	2338.801	224	0.904	400,015.917	0.044	0.050 (0.049 0.052)		
metric	2381.928	237	0.902	399,935.042	0.045	0.049 (0.048 0.051)	−0.002	−0.001
scalar	2983.313	250	0.876	400,540.587	0.051	0.054 (0.053 0.056)	−0.026	+ 0.005
Partial scalar	2450.741	246	0.900	399,918.960	0.045	0.049 (0.047 0.051)	−0.002	0.000
**clinical**								
configural	341.598	226	0.924	18,797.268	0.060	0.057 (0.044 0.069)		
metric	365.170	239	0.917	18,746.046	0.072	0.058 (0.046 0.070)	−0.007	+ 0.001
scalar	393.436	252	0.907	18,700.746	0.074	0.060 (0.048 0.071)	−0.010	+ 0.002
strict	397.285	269	0.907	18,262.298	0.075	0.056 (0.044 0.067)	0.000	−0.004

*Note:* Measurement invariance across sex analysis based on our newly explored structure (17 items and four factor structure).configural, configural invariance; metric, metric invariance; scalar, scalar invariance; partial scalar, partial scalar invariance; strict, error variance invariance; X^2^, Chi-square; *df*, degrees of freedom; CFI, comparative fit index; RMSEA, root-mean-square error of approximation; SRMR, standardized root mean squared residual; BIC, Bayesian information criterion

## Discussion

In the current study, a newly established four-factor structure was affirmed as the most suitable factor structure for a Chinese version of the 18-item TEPS in both clinical and non-clinical samples. In this four-factor structure, anticipatory pleasure and consummatory pleasure factors were each subdivided based on a motivation dimension. Measurement invariance analysis across clinical and non-clinical samples achieved strict invariance. Measurement invariance analysis across sex obtained strict invariance in the clinical sample and partial scalar invariance in the non-clinical sample.

In line with previous research, the TEPS was demonstrated to be a reliable, stable tool for assessing anhedonia in this study, with a Cronbach’α and McDonald’s omega > 0.80 and a retest coefficient of 0.70 [15, 24]. Furthermore, this study confirmed that the 18-item TEPS can be used to assess consummatory pleasure and anticipatory pleasure separately in Chinese respondents [15, 22]. Although item 11 and item 12 were transferred from anticipatory pleasure to consummatory pleasure in this study, the contents of these items contain a definite time and location, probing the ability of people to feel pleasure before anticipatory events have occurred. For example, item 11 (When I’m on my way to an amusement park, I can hardly wait to ride the roller coasters) suggests that being route to an amusement park can be enough to make someone feel pleasure. Consistent with previous research, we found that item 7 had low factor loading and thus decided to omit it in our CFA [19, 24]. A prior TEPS study in China also reported that item 7 had weak relations to others items and argued that there may be a cultural divergence in the interpretation of item 7 [24].

The consummatory-anticipatory division of anhedonia has gained substantial empirical evidence in animal studies as well as in human neurophysiological and behavioral studies [12, 56, 57]. There appears to be dissociable neural substrates mediating consummatory pleasure and anticipatory pleasure, with the former having been related to opioid neurotransmission onto GABAergic spiny neurons in the nucleus accumbens and the latter being related to mesolimbic dopaminergic neurotransmission [12]. Parsing anhedonia into finer components will benefit our understanding of the role of specific neurotransmitters and neural systems in mediating the psychological phenomenon of anhedonia. In addition, the full measurement invariance affirmed here across sex groups as well as across clinical versus non-clinical samples supports the view that the TEPS can be considered a stable assessment tool for the evaluation of consummatory pleasure and anticipatory pleasure. Furthermore, our comparison of goodness-of-fit indices among the initial two-factor structure of the TEPS [15], a previously published four-factor structure for a Chinese version of the TEPS with two added items [24], and the presently introduced four-factor structure that was newly developed through CFA indicates that our newly developed four-factor structure provides an improved factor structure in both non-clinical and clinical samples.

Regarding our division between with and without motivation-driven dimensions of consummatory and anticipatory pleasure, the role of motivation in anhedonia may involve value and effort computation for goal-directed behaviors, which are key in reward processing [17, 58]. Regarding motivation deficits related to anhedonia, someone who does not enjoy a typically rewarding activity due to anhedonia would be expected to lack motivation to pursue that activity in the future [59]. In turn, an individual with motivation deficits may experience less pleasure, favoring a vicious cycle between a motivation deficit and anhedonia. For example, compared to healthy controls, people with MDD tend to choose lower effort-expenditure rewarding tasks and expend less effort in a progressive ratio task [60]. Similarly, people with schizophrenia have been found to also tend to favor lower effort-expenditure rewarding tasks than controls, and their anhedonia symptoms have been shown to correlate with motivation deficit magnitude [61, 62]. Hence, it is important to have a scale that can be used to assess motivation in the context of anhedonia, and the presently developed four-factor structure of the TEPS suggests that the TEPS has the potential to be used in such an application.

The first factor identified, namely anticipatory pleasure with motivation-driving, reflects the need for approach motivation to achieve anticipated events, including making plans and anticipating effort (e.g., item 14, When I think of something tasty, like a chocolate chip cookie, I have to have one). Conversely, the second factor, anticipatory pleasure without motivation-driving, encompasses anticipatory events but with a limited need for motivation or an affirmative attitude (e.g., item 15, Looking forward to a pleasurable experience is in itself pleasurable). Meanwhile, the third factor, consummatory pleasure with motivation-driving, refers to pleasurable experiences in which one must be motivated to actively remember a pleasure to activate and maintain a representation (e.g., item 9, I love it when people play with my hair). Lastly, the fourth factor, consummatory pleasure without motivation-driving, refers to immediately accessible enjoyment without a need to desire more. Constituent items of this fourth factor tend to refer to natural events involving limited subjective initiative (e.g., item 13, I appreciate the beauty of a fresh snowfall).

The motivation dimension affirmed in our analyses was found to exist for both consummatory pleasure and anticipatory pleasure, a supposition that is supported by previous empirical evidence. For example, consummatory and anticipatory pleasure were found to be related to motivation-associatedevent-related potential signals in a study employing a cue gambling task [63]. Meanwhile, significantly impaired consummatory and anticipatory pleasure in schizophrenia may only emerge in the context of a serious motivation deficit [64]. Hence, the consideration of motivation in the factor structure of the TEPS may provide new insight enabling a more comprehensive understanding of the TEPS, thus expanding the applicability of the TEPS in relation to a more detailed structural understanding of anhedonia.

The newly explored four-factor structure showed good convergent validity and discriminate validity. Previous studies mainly focused on the initial two-factor structure’s convergent and discriminate validity through correlation analysis. In the initial study, Grad et al. demonstrated that the two-factor structure have good convergent validity and discriminate validity. The result of correlation analysis indicated that both anticipatory pleasure factor and consummatory pleasure factor significantly correlated with other scales measuring anhedonia and showed separation correlation patterns as well [15]. Subsequently, Paul et al. not only demonstrated that the two-factor structure have good convergent validity and discriminate validity, but also indicated that TEPS can possibly assess motivation components of anhedonia [19]. Although prior studies only demonstrated that the two-factor structure showed good convergent validity and discriminate validity, this study also showed that the newly explored four-factor structure has good convergent validity and discriminate validity through CR and HTMT indexes.

The present findings of full measurement invariance (configural, metric, scalar, and error variance) for our newly explored four-factor structure across clinical and non-clinical samples indicate that differences in TEPS performance between the clinical and non-clinical respondents can be interpreted reliably. This affirmation is important given that TEPS scores have been reported to differ between clinical and non-clinical groups and appear to be differentially affected across clinical groups (e.g. impaired anticipatory pleasure and consummatory pleasure in MDD [32], but bias toward impairment in anticipatory pleasure in schizophrenia [20, 29–31]). Furthermore, recent studies employing reward task-choice behavioral analyses have suggested that there may be distinct clinically important motivation impairments in relation to anhedonia in patients with schizophrenia, bipolar disorder, and MDD [65, 66]. Hence, measurement invariance provides confidence that TEPS score differences reflect authentic differences across the well-establishedanticipatory-consummatory pleasure dimension as well as across a newly exposed motivation-nonmotivation dimension in future research.

With respect to measurement invariance across sex in a non-clinical sample, based on modification indices, we permitted correlations of the error variance between item 2 (The sound of crackling wood in the fireplace is very relaxing) and item 4 (I love the sound of rain on the windows when I’m lying in my warm bed). These two items both contribute to the consummatory pleasure without motivation-driving subscale and reflect pleasure related to a natural phenomenon, which may be related to a residual correlation between them. The intercepts of items 2, 9, 11, and 18 were free estimated when we conducted scalar invariance analysis, indicating that researchers should further test whether these items perform differently in relation to sex in a non-clinical sample. Similarly, Zhou et al. also reported partial scalar invariance across sex in an undergraduate sample employing a 20-item version of the TEPS [27]. Notwithstanding, the full measurement invariance observed across sex in our clinical sample may be more important in the context of exploring the mechanisms of anhedonia. With respect to sex differences in anhedonia [34, 35], some researchers have reported that men are more likely to experience anhedonia than women [67]. Indeed, a meta-analysis indicated that, compared to females, males with schizophrenia spectrum disorders report lower anticipatory pleasure and consummatory pleasure, as measured by the TEPS [30]. Overall, affirmation of measurement invariance provides a premise for probing differences in anhedonia pattern between males and females.

Some limitations of this study should be noted. First, because this study was conducted in a Chinese cultural context, the generalizability of the findings to other cultural contexts remains to be determined. Using a 20-item version of the TEPS in China, Chan et al. did find full measurement invariance for consummatory pleasure and anticipatory pleasure subscales across cultures, which may offset this limitation to some [68]. Second, our enrollment of college students as a non-clinical sample may restrict the generalizability of our findings to other age ranges. With respect to age generalization, however, prior studies examining the TEPS in community samples have affirmed its stability across age bands [21, 23]. Third, the cut-off points of indices used for CFA and TLI may be too lax for the assessment of the goodness of fit in this study, as recently some researchers proposed considered values over .95 for CFI and TLI and below .08 for SRMR and .06 for RMSEA [49], or values above .95 or .97 for CFI and TLI paired with values lower than .05 for SRMR and RMSEA as representative of a good fit with data [69]. Even Sivo et al. considered sample size to establish cut-off points [70], which means that depending on the sample size of this study, values higher than .99 for CFI and TLI accompanied by values lower than .05 and below .03 for RMSEA are indicative of a good fit to data. However, it also should be noticed that the fit indices of new four-factor found in this study achieved traditional criteria of the goodness of fit, and better than others two models. Lastly, as Fan and Sivo suggested, the Δgoodness-of-fit indexes are designed to assess model fit in terms of covariance structure and may be sensitive to model size [71], which should be considered especially when mean structure invariance is the research focus.

## Conclusions

For the first time, this study determined the most suitable factor structure for a Chinese version of the 18-item TEPS with multiple methods and further confirmed the resultant four-factor structure in clinical and non-clinical samples. Besides, the newly four-factor structure was demonstrated with good convergent validity and discriminate validity. Full measurement invariance was observed between clinical and non-clinical samples and between sex in a clinical sample. Partial scalar invariance was found across sex in a non-clinical sample. The present results provide insights into the psychometric properties of the TEPS as well as the psychological phenomenon of anhedonia.

## Supplementary Information

**Additional file 1.** Supplementary Table 1 Goodness-of-fit indices obtained for compared structural models of the TEPS in clinical sample (sample 3).

## Data Availability

The datasets generated and analyzed during the current study are not publicly available due to no permission from participants to share anonymized participant data publicly but are available from the corresponding author on reasonable request. This study got permission from the original creators of the TEPS to use and translate their work in this way.

## Abbreviations

TEPS: the Temporal experience of Pleasure Scale
EFA: exploratory factor analysis
CFA: confirmatory factor analysis
MIC: mean inter-item correlation
MAP: Velicer’s minimum average partial
MLR: maximum likelihood with robust standards errors
X^2^: Chi-square
df: degrees of freedom
CFI: comparative fit index
TLI: Tucker-Lewis index
RMSEA: root mean square error of approximation
