# Supplementary material for: Factor structure and sex invariance of the temporal experience of pleasure scale (TEPS) in Chinese university students and clinical population
Source: BMC Psychiatry. 2021 Jul 28;21:378. doi: 10.1186/s12888-021-03379-9 (PMC8317394; doi:10.1186/s12888-021-03379-9)
Supplement: Supplementary file 1 — Additional file 1. Supplementary Table 1 Goodness-of-fit indices obtained for compared structural models of the TEPS in clinical sample (sample 3). [file 12888_2021_3379_MOESM1_ESM.docx]

**Supplementary material**

Supplementary table 1

Goodness-of-fit indices obtained for compared structural models of the TEPS in clinical sample (sample 3)

|  | X^2^ | *df* | CFI | TLI | SRMR | RMSEA (90%CI) |
| --- | --- | --- | --- | --- | --- | --- |
| Model1 | 282.427 | 134 | 0.890 | 0.875 | 0.061 | 0.059(0.050 0.069) |
| Model2 | 211.317 | 113 | 0.924 | 0.909 | 0.054 | 0.053(0.042 0.064) |
| Model3 | 173.014 | 113 | 0.954 | 0.944 | 0.048 | 0.041(0.028 0.053) |
| Second-level | 178.023 | 114 | 0.951 | 0.941 | 0.049 | 0.042(0.030 0.054) |

*Note:* Model 1 is the two-factor structure proposed by Gard(18 items and two factor structure) [1]. Model 2 is the four-factor structure proposed by Chan [2] with two added items, and item 7 regarded as expendable(20 items and four factor structure). Model 3 is our newly developed four-factor structure without item 7(18 items and four factor structure). X^2^, Chi-square; df, degrees of freedom; CFI, comparative fit index; TLI, Tucker‐Lewis index; SRMR, standardized root mean squared residual; RMSEA, root mean square error of approximation.

Reference:

[1]Gard DE, Gard MG, Kring AM, John OP. Anticipatory and consummatory components of the experience of pleasure: A scale development study. Journal of Research in Personality. 2006;40:1086-102. https://doi.org/10.1016/j.jrp.2005.11.001

[2]Chan RCK, Shi Y-f, Lai M-k, Wang Y-n, Wang Y, Kring AM. The Temporal Experience of Pleasure Scale (TEPS): Exploration and Confirmation of Factor Structure in a Healthy Chinese Sample. Plos One. 2012;7. https://doi.org/10.1371/journal.pone.0035352
